# Attachment insecurities, emotion dynamics and stress in intimate relationships during the transition to parenthood

**DOI:** 10.1186/s40359-024-01686-w

**Published:** 2024-04-12

**Authors:** Georgia Kouri, Nathalie Meuwly, Marianne Richter, Dominik Schoebi

**Affiliations:** University of Fribourg, Department of Psychology, Rue de Faucigny 2, 1700 Fribourg, Switzerland

**Keywords:** Attachment insecurity, Emotion dynamics, Emotional inertia, Intimate relationships, Stress, Parenthood

## Abstract

**Background:**

In intimate relationships, which are characterized by emotional interdependence, partners act as attachment figures which serve emotion regulation functions. The experience of emotions as well as the strategies that partners use to regulate them and to respond to relational experiences, especially during stressful periods, differ greatly according to their attachment orientation. An important aspect in emotion dynamics is emotional inertia, which reflects the degree to which a person’s current affective state is resistant to change on a moment-to-moment basis. Inertia has been related to maladaptive emotion regulation strategies, like suppression and rumination, preferentially used by highly anxious and avoidant individuals. The aim of this study is to examine associations between attachment orientations and reports on the experience of positive and negative affect, and their dynamics in daily life across the transition to parenthood.

**Methods:**

Longitudinal data from a sample of 152 mixed-gender couples collected across the transition to parenthood was analyzed. We predicted that individuals with a more insecure attachment would report more negative and less positive affect, and that their emotional experience would be more resistant to change over time. We explored effects when participants reported feeling stressed.

**Results:**

The data suggested that attachment anxiety was associated with less positive and more negative affect and that attachment avoidance was associated with more positive affect. Anxious individuals showed lower emotional inertia and not higher as we expected. Reported stress for anxious and avoidant individuals was significantly associated with more negative but not less positive affect.

**Conclusions:**

Results are discussed in the light of their impact on couples during stressful periods. Differences between anxiety and avoidance are found, emphasizing the importance of attachment insecurities on the experience of emotion. Furthermore, our findings on momentary fluctuating affect offer complementary insight into the emotional functioning of individuals with different attachment orientations.

## Introduction

Emotions are a central aspect of how individuals experience their intimate relationships [1]. They help signaling and communicating personal needs and concerns to oneself and to the partner [2, 3]. Emotions are not static. Rather, they emerge and dissolve across time [4] and their expression and experience during interactions with intimate partners are predictive of relationship functioning and development [5]. The factors that shape emotional experience in intimate relationships are therefore important for individual well-being and relationship functioning, particularly during important life transitions that challenge couples, such as the transition to parenthood. An important factor influencing both emotion regulation and relationship functioning is adult attachment orientation [6]. When individuals enter new relationships, they bring with them so-called internal working models of relationships– including assumptions and expectations about how people behave in relationships– shaped by their history of interpersonal experiences [7]. Such attachment orientations guide relationships [8], and shape how individuals feel about and respond to their intimate partners [9]. Differences in attachment orientations affect patterns of emotional experience, as well as the strategies individuals use to regulate their emotions, especially when dealing with stress [10]. Attachment orientations thus, should shape daily emotional experiences in intimate relationships [11]. The current study examines this possibility during the transition to parenthood, examining the link between attachment orientations, momentary emotional experiences and stress.

In the remainder of the introduction, we first discuss the literature on emotion and emotion dynamics as related to individual and relational functioning. We then discuss attachment orientations, and how they may shape the experience of emotions in intimate romantic relationships.

### Emotion dynamics

Emotions are characterized by frequent change [12]. They fluctuate over time and are thought to be continuously regulated to optimally fit with the current desired state [13]. People differ greatly in their emotional responses to environmental challenges [14], and some individuals show a relatively high degree of resistance to change of their emotions, commonly referred to as emotional inertia [15]. High emotional inertia reflects a pattern of emotions characterized by a high degree of moment-to-moment stability of emotions, with emotions being resistant to external (changes in the environment) and internal (regulation efforts) influences. Low emotional inertia reflects a pattern of emotions characterized by high moment-to-moment instability of emotions [15]. High emotional inertia may reflect a lack of emotion regulation [16], such that emotions carry over from one moment to the next, possibly because people tend to perceive and interpret the world around them in ways congruent with their current affective state [13]. Some possible explanations have been proposed for the occurrence of static emotions. For example, emotional inertia may result from the inability to regulate emotions in an efficient way even if there is a motivation to do so [17]. Also, exposure to intense events eliciting strong emotional reactions and difficulty downregulating elicited emotions, can result in an increased emotional dependency over time [18]. Studies suggest that elevated inertia not only of negative, but also of positive emotion is reliably associated with poorer well-being [15, 19]. Specifically, emotional inertia of both positive and negative emotions was associated with low self-esteem and depression [15]. Although these findings need further examination and replication, they point to the possibility that positive emotions that are slow to change across contexts may also be reflective of an affective system with limited responsiveness to environmental cues, possibly hampering regulatory processes [20]. Inert patterns of emotion may point to a lack of emotional flexibility and restrict an individual’s capacity to actively respond to situational challenges across varying conditions. They have also been associated with poorer psychological adjustment [21], impaired social functioning [22], and lower relationship quality [23]. Highly inert emotion patterns over time may therefore serve as a relevant proxy of maladjustment in everyday life.

### Attachment and emotion in romantic relationships across the transition to parenthood

Attachment theory provides a framework for explaining how early relational dynamics with caregivers add to emotional stability in adulthood [24]. In intimate relationships, the partner or spouse represents an important attachment figure [25] and when people face challenges and feel stressed, they often attend to their closest relations to maintain or re-establish security and comfort [26]. They seek emotional or instrumental support from their romantic partners [27] although the specific strategies people will use to achieve this goal vary as a function of their attachment history [26].

Individuals with a secure attachment orientation can rely on others during times of need [28], they attribute less hostile intent to others when tensions arise [29] and they experience more positive emotions in their relationships [30]. Individuals with an anxious attachment orientation tend to focus on negative emotions [11] and overemphasize their helplessness [31]. They experience sadness more frequently [6] and report less positive emotions in the relationship [30]. Furthermore, they appear to be more distressed [32], ruminate, and harbor more anger [29], probably resulting from the expectation that people are unpredictable and inconsistent [24]. Individuals with an avoidant attachment orientation mask or inhibit expressions of emotion [11], to avoid appearing vulnerable and experiencing further rejection-related distress [33]. They tend to believe that their needs will not be met in a particular relationship [24], so they minimize experiences of negative affect [34, 35], and engage in emotional withdrawal [36]. At the same time, individuals higher in avoidance orientation, have also been found to report more frequent negative and fewer positive emotions in their relationship [30, 37, 38], more intense negative emotional reactions after the occurrence of negative events [39], more intense physiological recordings of anger, as well as higher cortisol levels during conflict [40].

The transition to parenthood constitutes a major challenge in one’s life and is for many marked by periods of stress and emotional turmoil, which can negatively impact relationship quality [41–44]. Parenthood brings new roles and responsibilities for mothers and fathers, and both partners need to adjust to a multitude of changes and stressors during the transition to parenthood [45]. Indeed, such changes in daily life tend to contribute to higher levels of stress during the transition to parenthood [41–44, 46]. As a result, these changes may undermine personal and relational well-being [47]. It may well be a time when attachment processes are particularly activated [48], and how the becoming parents adjust to this transition is shaped by their attachment orientation. As a consequence, their relationships will also differ in their emotional tone [30]. Specifically, highly anxious women enter the transition to parenthood perceiving lower levels of spousal support, leading to declines in marital satisfaction [49] and increases in depressive symptoms [50]. Parents higher in avoidance report more difficulties in adjusting to parenthood [51] and higher parenting stress [52].

In sum, the literature underscores the importance of attachment orientations for individual adjustment, shaping the experience of emotions in intimate relationships [53]. Individual differences in attachment orientations are reflected in different perceptions of oneself and others, shaping the experience of emotions in intimate relationships, particularly during times of stress, like the transition to parenthood.

### Attachment, emotion, and emotion dynamics in romantic relationships

Research has indicated that individuals exhibit variations in their average levels of positive and negative emotions [54], highlighting how people are distinguished based on their typical emotional experiences. Particularly, people differ widely in their preferences for the strategies they use to regulate their own and their partner’s emotions. For instance, rumination, a maladaptive process involving repetitively thinking about negative emotions [55] was found to be associated with emotional inertia, and individuals with an anxious attachment orientation tend to use this strategy more than their securely attached counterparts [21, 56]. The stronger and more persistent repetitive thoughts occur on a particular day, the greater the impact on affective states that persist across different occasions [57] and result in intense negative feelings, such as anger [58], and in distressed close relationships [59, 60].

Suppression of emotion expression is another maladaptive emotion regulation strategy that has been linked to emotional inertia. Individuals with an avoidant attachment orientation tend to use suppression more than individuals with a secure or anxious attachment orientation [56, 57] probably for self-protective reasons [33]. Suppression does not alleviate negative emotions [61]. Indeed, individuals who tend to use suppression as a strategy report lower levels of intimacy and more negative emotional experiences [9, 62].

Overall, a high degree of emotional inertia may mirror a lack of emotional flexibility [12] and is associated with emotion regulation strategies preferentially used by anxious and avoidant individuals [63]. Therefore, since individuals high in anxiety and avoidance use emotion regulation strategies associated with emotional inertia, this study examines whether people exhibiting higher levels of insecurity will experience more negative and less positive affect and also exhibit higher emotional inertia.

### The current study

The objective of this study was to investigate potential associations between attachment orientations and distinct patterns of momentary emotional experiences, emotional responses and emotional inertia in a daily life context during the transition to parenthood. The exploration of the relationships between attachment orientations and individuals’ emotional experiences, responses, and the dynamic patterns of change in daily life is of considerable significance because both insecure attachment orientations and emotional dynamics have previously been correlated with psychological maladjustment and mental health issues [15, 49].

The attachment system is activated in stressful and threatening instances helping to regulate stress that is associated with perceived threat by controlling proximity to the attachment figure [26, [7]–in the case of intimate relationships, the partner. In other words, the attachment system serves stress and emotion regulation purposes, and therefore the effects of an insecure attachment style should become apparent at the level of emotional responses and dynamics when people experience stress. Having a baby can be a joyful but also chronically stressful experience and attachment insecurities make individuals more vulnerable to personal and interpersonal problems during this time [64]. Bowlby (1988) [65] believed that the transition to parenthood should be a critical period for systematic changes in the attachment system.

The transition to parenthood is a major life transition characterized by challenges that often affect relationships [45] sometimes leading to marked decreases in marital satisfaction and personal well-being [66]. Highly anxious women enter the transition to parenthood perceiving less spousal support and with steeper declines in their marital satisfaction [66] prior to the baby’s birth [67]. It has also been found within a sample of couples expecting their first baby, that partners experience more negative affect due to increased stress [45], more negative perceptions, and decreases in relationship satisfaction [68]. We assessed individuals’ emotional experiences in daily life using a momentary assessment approach during the transition to parenthood [69].

We hypothesized that individuals higher in attachment anxiety (H1a) and individuals higher in avoidance (H1b) would generally experience less positive and more negative affect. On more exploratory grounds, we further specified this hypothesis such that individuals higher in attachment anxiety and avoidancewould report more negative and less positive emotional experiences than their more secure counterparts, in response to momentary stress experiences (i.e. when they reported feeling stressed). Finally, we hypothesized that individuals with higher levels of anxiety (H2a), as well as individuals with higher levels of avoidance (H2b) would exhibit more inert positive and negative emotional experiences than relatively secure individuals.

## Method

### Participants

Participants were recruited from flyers and word-of-mouth advertisement. Eligible couples had to speak the study languages, be over 18 years old, live in the same household and expecting their first child. The current sample consisted of 152 mixed-gender couples (*n* = 304). The mean age was 31.55 for women (*SD* = 3.66) and 33.19 for men (*SD* = 4.06). The mean relationship duration was 6.73 years (*SD* = 3.01). The sample size was determined before recruitment started, based on a priori power estimation of a medium sized between-subject effect (effect size *r* =.25) on a within-subject slope, which suggested statistical power of 0.8 for 286 participants from 143 couples.

### Procedure

The data for this study are part of a longitudinal study with four measurements (pregnancy, 6, 12 and 18 months after the birth of the first child). The data used for the current analyses were collected at the first measurement - in the second or third trimester of pregnancy. First, each participant completed an online survey that included questions on demographic characteristics, mental health, well-being, interpersonal dispositions, attachment orientation, and on evaluations of the relationship. Second, participants completed a smartphone-based momentary assessment procedure during seven consecutive days, four times per day (8h00, 12h00, 18h00 and 21h30). It included sets of questions related to participants’ momentary emotional states and interpersonal experiences. Participants received a message in order to complete the assessment at each time point of the day and they could complete it in the next ninety minutes. Before starting the daily assessment, all participants were provided with detailed instructions on the use of the momentary assessment. Other assessments of the study included a diagnostic telephone interview on mental health, home visits with interaction tasks, physiological measures and an evaluation week, which included a three-day assessment of physiological measures and the seven-day momentary assessment. All participants completed and signed the informed consent form and after each assessment point, they received approximately 180 $ per couple. The project was approved by the ethics review board of the regional government. The study and hypotheses were not pre-registered.

### Measures

#### Attachment orientation

The Experiences in Close Relationships-Revised [70] French version [71] and German version [72] was used to assess individual attachment on two dimensions: anxiety and avoidance. The questionnaire includes 36 items, of which 18 assess attachment anxiety, the degree to which individuals feel insecure about the availability and responsiveness of their partner (e.g., I need confirmation that my partner loves me; females: α = 0.867; males: α = 0.878), and another 18 items assess attachment avoidance, the degree to which individuals avoid closeness and intimacy (e.g., I try to avoid getting too close to my partner; females: α = 0.873; males: α = 0.815). Participants responded to each item using a 7-point scale from 1 (*strongly disagree*) to 7 (*strongly agree*), rating the extent to which each item is descriptive of how they usually feel and behave in romantic relationships. Ratings were averaged to compute scores for each dimension. Higher scores reflected more anxious and more avoidant attachment orientations.

#### Emotional states

Emotions were assessed by daily diaries and participants rated the degree to which they felt “happy”, in a “good mood”, “depressed”, “irritated”, and “worried”. A continuous slider on a 10-point-scale was used, ranging from 1 (*not at all*) to 10 (*extremely*). The ratings of the items “happy” and “good mood” were averaged to form a Positive Affect (PA) measure and the two items were highly consistent within person (Ω = 0.858) and between individuals (Ω = 0.976). The ratings of the items “depressed”, “irritated”, and “worried”, were averaged to reflect negative affect (NA). This momentary NA measure showed satisfactory internal consistency within person (Ω = 0.652) and between individuals (Ω = 0.853). Between individuals internal consistency was high for PA (females: α = 0.965; males: α = 0.969) and for NA (females: α = 0.853; males: α = 0.836).

#### Emotional inertia

Emotional inertia is defined as resistance to emotional change, commonly formalized as the degree to which a person’s current emotional state can be predicted by his or her emotional state at a prior moment. Thus, high emotional inertia suggests that a person’s current emotional experience is likely to persist from one moment to the next and compromise an individual’s capacity to respond adaptively across varying conditions and demands. On the contrary, moderate emotional inertia indicates that a person’s emotional states are more changeable across time [15]. Low levels of emotional inertia may indicate emotional instability and undermine adaptive behavior [73]. Emotional inertia as addressed in this study, reflects an important aspect of a person’s emotion dynamics across hours in daily life, and is operationalized as the first-order autocorrelation among repeated measures of emotional states.

#### Stress

Stress was assessed by participants’ daily ratings of the degree to which they felt stress at a specific moment (“Currently, how stressed do you feel?”). A continuous slider on a 10-point-scale was used to measure the extent that participants felt stressed, ranging from 1 (*not at all*) to 10 (*extremely*).

### Data analysis

We used a mixed modelling approach to analyze the data, as it allows to model dependencies of individuals’ repeated reports within time points and couples. Our sample included mixed-gender couples, so dyads’ members were distinguishable by their reported gender [74]. However, model comparisons suggested a better fit for models with pooled estimates instead of separate coefficients for the two genders (likelihood ratios > 540.795, df = 16, *p* <.001), and we therefore present models with pooled estimates.

We set up a dyadic model that captures both partners’ reports sampled at the same time points repeatedly and clustered within couples. Models were run with the package nlme [75] in R Studio [76]. Level 1 predictors (lagged emotion and stress) were centered at the person mean and Level 2 predictors (anxiety and avoidance) were centered at the grand mean. We estimated random intercepts and allowed for random variation of lagged emotion slopes and stress slopes across couples.

We ran two different models, one predicting PA reports and one predicting NA reports, to test whether attachment orientations predicted emotional states, and also emotional inertia and fluctuation of emotional states as a function of stress reports. Specifically, we ran models testing anxiety (H1a) and avoidance (H1b) as predictors of current PA and NA. We also included interactions with stress reports to test whether anxiety and avoidance predicted positive and negative affect as associated with stress reports. And to test our predictions that anxiety (H2a) and avoidance (H2b) were associated with greater emotional inertia, we included interaction effects between attachment anxiety and attachment avoidance and lagged PA reports and between attachment anxiety and avoidance and lagged NA reports respectively, as predictors of concurrent PA or NA.

Equation 1 shows the model, examining within person fluctuations of PA or NA as a function of lagged PA or NA, and stress, as associated with attachment anxiety and avoidance (and adjusted for a general trend across repeated measurements). Within person predictors: The coefficient for lagged PA or NA (π_*1i*_) reflects the extent to which the current emotional state is a function of the emotional state reported at the prior measurement (t-1), and therefore represents emotional inertia, while the coefficient for stress (π_*2i*_) reflects the extent to which stress reports predicted less positive or more negative emotional states. The coefficient for π_*3ti*_ captures the general trend in PA or NA across the reporting period.

1 $$\eqalign{{\rm{Emotio}}{{\rm{n}}_{ti}}\,{\rm{ = }} & {{\rm{\pi }}_{0i}}\,{\rm{ + }}\,{{\rm{\pi }}_{1ti}}\,\left( {{\rm{emotio}}{{\rm{n}}_{\rm{t}}}{{\rm{ - }}_{{\rm{1i}}}}} \right)\,{\rm{ + }}\,{{\rm{\pi }}_{2ti}}\,\left( {{\rm{stress}}} \right) \cr & {\rm{ + }}\,{{\rm{\pi }}_{3ti}}\,\left( {{\rm{trend}}} \right)\,{\rm{ + }}\,{{\rm{\pi }}_{4i}}\,\left( {{\rm{Anxiety}}} \right) \cr & {\rm{ + }}\,{{\rm{\pi }}_{5i}}\,\left( {{\rm{Avoidance}}} \right)\,{\rm{ + }}\,{{\rm{\pi }}_{6i}}\left( {{\rm{Anxiety}}\, * \,{\rm{stress}}} \right) \cr & {\rm{ + }}\,{{\rm{\pi }}_{7i}}\,\left( {{\rm{Avoidance}}\, * \,{\rm{stress}}} \right)\,{\rm{ + }}\,{{\rm{\pi }}_{{\rm{8}}i}}\,\left( {{\rm{Anxiety}}\, * \,{\rm{emotio}}{{\rm{n}}_t}{{\rm{ - }}_{1i}}} \right) \cr & {\rm{ + }}{{\rm{\pi }}_{9i}}\,\left( {{\rm{Avoidance}}\, * \,{\rm{emotio}}{{\rm{n}}_t}{{\rm{ - }}_{1i}}} \right){\rm{ + }}{{\rm{e}}_{ti}} \cr} $$

Between-person predictors and interaction terms: The coefficients π_*4i*_ and π_*5i*_ capture the associations between anxiety or avoidance and individual differences in average PA and NA reports. The coefficients for π_*6i*_ and π_*7i*_ are interaction effects between attachment orientations and stress, capturing the extent to which anxious or avoidant attachment orientations are associated with the stress effects on PA or NA. The coefficients for *π*_*8i*_ and *π*_*9i*_ represent interaction effects between lagged PA or NA and attachment anxiety and avoidance. These coefficients represent moderator effects of the first order autoregressive effects of positive or negative affect across repeated measurements. Positive coefficients would indicate that prior affect reports of more anxious or avoidant individuals would more strongly and positively predict subsequent affect reports, which can be interpreted as PA or NA inertia being stronger in more anxious or avoidant individuals. The error term e_*ti*_ denotes the residual variance.

First, we ran models with separate coefficients for female and male participants. Next, we compared the fit of these models with that of models in which we imposed equality constraints for female and male participants’ coefficients for those coefficients relevant to test the hypotheses. These models comparisons yielded superior AIC and BIC indices for all models with constraints and suggested that estimating separate parameter estimates for men and women did not improve the model fit significantly (χ^2^ (2)[] > 3.001, *p* >.223). As a result, we report models with pooled estimates.

## Results

### Descriptive statistics

Table 1 presents the means *(M)* and the standard deviation (*SD)* for all our variables for women and men. Correlation coefficients among study variables are presented in Table 2. Across and within individuals *M* and *SD*s were calculated. Specifically, between all individuals, the average report of positive affect was *M* = 7.59 (*SD* = 1.73), the average report of negative affect was *M* = 1.06 (*SD* = 1.41) and the average report of stress was *M* = 1.74 (*SD* = 2.19). The within person variability was *SD* = 1.38 for positive affect, *SD* = 1.08 for negative affect and *SD* = 1.68 for stress. The intraclass correlation (ICC) for positive affect was 0.824 (CI 0.817 − 0.832) and for negative affect it was 0.462 (CI 0.447 − 0.476).

**Table 1:** Participants mean ratings of attachment orientation, stress and emotional states

	Women		Men				
	Means	SD	Means	SD	t-test		Cohen’s d
Anxiety	2.73	0.73	2.65	0.76	0.118	0.906	0.350
Avoidance	4.59	0.37	4.55	0.34	− 0.657	0.512	0.167
Stress	1.49	2.08	2.02	2.26	0.363	0.717	0.816
PA	7.59	1.86	7.59	1.73	0.123	0.902	0.723
NA	0.84	1.34	1.06	1.41	0.866	0.388	0.771
PA lagged	7.58	1.87	7.58	1.73	− 0.221	0.826	1.113
NA lagged	0.84	1.34	1.07	1.41	-2.659	0.009	1.076

*Note* PA = Positive Affect; NA = Negative Affect

**Table 2:** Correlation Matrix for Anxiety, Avoidance, Stress, PA, NA, PA-lagged, NA lagged

	1.	2.	3.	4.	5.	6.	7.
1.Anxiety	-						
2.Avoidance	− 0.135**	-					
3.Stress	0.137**	− 0.037*	-				
4.PA	− 0.155**	0.100**	− 0.479**	-			
5.NA	0.199**	− 0.027	0.573**	− 0.555**	-		
6.PA-lagged	− 0.155**	0.101**	− 0.357**	0.586**	− 0.360**	-	
7.NA-lagged	0.193**	− 0.020	0.417**	− 0.357**	0.569**	− 0.553**	-

**. Correlation is significant at the 0.01 level (2-tailed)

*. Correlation is significant at the 0.05 level (2-tailed)

### Attachment predicting daily affect

We predicted that both attachment anxiety (H1a) and avoidance (H2b) were associated with less PA and more NA reports. Overall, we found significant associations between anxiety, and PA and NA respectively, supporting H1a and partially supporting H1b (see Table 3). Specifically, anxiety was significantly and negatively associated with PA (*b* = − 0.335, *p* =.000), and positively with NA (*b* = 0.217, *p* =.000). Attachment avoidance was significantly associated with higher PA (*b* = 0.159, *p* =.043) but not with NA (*b* = − 0.064, *p* =.323; see Table 3).

**Table 3:** The effects of attachment anxiety, avoidance and stress on emotional states and lagged affect

		PA			NA	
	b	SE	*p*	b	SE	*p*
LA	0.004	0.204	0.98	0.227	0.234	0.332
Anxiety	− 0.335***	0.041	0.000	0.217***	0.034	0.000
LA x Anxiety	− 0.055***	0.018	0.000	− 0.003	0.019	0.842
Stress	− 0.273***	0.012	0.000	0.287***	0.010	0.000
Anxiety x Stress	− 0.023	0.016	0.162	0.078***	0.014	0.000
Avoidance	0.159*	0.079	0.043	− 0.064	0.065	0.323
LA x Avoidance	0.069	0.041	0.092	− 0.016	0.047	0.721
Avoidance x Stress	− 0.019	0.387	0.614	0.088**	0.032	0.006

*Note* * *p* <.05. ** *p* <.01. *** *p* <.001. PA = Positive Affect; NA = Negative Affect; LA = Lagged Affect

Analyses for our exploratory hypotheses on stress suggested that momentary stress significantly predicted more NA but not less PA (cf. Table 3). Furthermore, regarding differences of stress effects on emotional states, the results were in line with our expectations for NA only. Attachment anxiety did not significantly interact with stress to predict PA (*b* = − 0.023, *p* =.162) but it significantly predicted the experience of more negative emotion (*b* = 0.078, *p* =.000). The same pattern emerged for avoidance. Avoidance did not significantly interact with stress to predict positive emotion (*b* = − 0.019, *p* =.614), but it did so predicting more negative emotion (*b* = 0.088, *p* =.006). Estimating simple slopes (using a tool proposed by Preacher, Curren & Bauer, 2006) [77] suggest that for individuals with low anxiety (1 SD below average), stress effects on NA were lower, but still significant (*b* = 0.191, *p* >.001), and they were substantial for individuals with 1 *SD* above average anxiety (*b* = 0.383, *p* >.001). Likewise, for individuals with moderate attachment avoidance (1 *SD* below average), stress effects on NA were somewhat lower (*b* = 0.239, *p* >.001), whereas they were stronger (*b* = 0.336, *p* >.001) in participants with elevated levels of attachment avoidance (1 *SD* above average). In summary, the results suggest that stress was more strongly associated with more negative emotional states for individuals with an anxious or avoidant attachment orientation, as compared to relatively secure individuals, but no equivalent differences emerged for stress associations with positive affect. To ensure that simple effects of attachment and lagged emotion are not affected by stress, we ran separate analyses where stress was not included as a predictor (cf. Table 4).

**Table 4:** The effects of attachment anxiety and avoidance on emotional states and lagged affect

		PA			NA	
	b	SE		b	SE	*p*
LA	0.072	0.214	0.736	0.014	0.255	0.954
Anxiety	− 0.348***	0.044	0.000	0.234***	0.036	0.000
LA x Anxiety	− 0.048***	0.019	0.013	0.032	0.020	0.115
Avoidance	0.160*	0.083	0.055	− 0.067	0.070	0.338
LA x Avoidance	0.059	0.043	0.169	0.022	0.051	0.663

*Note* * *p* <.05. ** *p* <.01. *** *p* <.001. PA = Positive Affect; NA = Negative Affect; LA = Lagged Affect

### Were affect reports of insecure participants more inert across time?

We predicted that individuals high in attachment anxiety and avoidance orientation would show more inert positive and negative affect reports (Hypotheses 2a and 2b, ). Only one significant association resulted (cf. Table 3), which was contrary to our hypothesis. The data suggested that attachment anxiety significantly moderated autoregressive effects of PA, reflecting inertia, such that in more anxious individuals, prior PA reports predicted current PA reports more negatively or less positively (*b* = − 0.055, *p* =.003). Specifically, estimating simple slopes suggest that an individual who scores 1 *SD* above average in attachment anxiety would have an autoregressive effect of PA of *b* = − 0.051, whereas an individual who scores 1 *SD* below average in attachment anxiety would have an autoregressive effect of *b* = 0.059, with none of these estimates reaching significance (*p* >.495). We found no significant association between attachment anxiety and NA inertia (*b* = − 0.003, *p* =.842). Likewise, attachment avoidance did not predict higher PA (*b* = − 0.069, *p* =.092) or NA (*b* = − 0.016, *p* =.721) inertia.

## Discussion

The goal of this research was to examine whether individuals with insecure attachment orientations differed in their daily affective experience from individuals with more secure attachment orientations and explored associations with stress. We expected that individuals high in attachment anxiety orientation (H1a) and individuals high in attachment avoidance orientation (H2b) experienced less positive and more negative emotional states, and that people higher in anxiety (H2a) and avoidance (H2b) would show elevated inertia in their positive and negative affect reports.

Overall, the data provided support for generally lower positive and higher negative affect reports for individuals with an anxious attachment orientation, supporting H1a. In contrast, attachment avoidance was not significantly associated with negative affect. Rather, a small positive association emerged for positive affect, suggesting that individuals high in attachment avoidance may actually experience more positive affect, contradicting our hypothesis H1b. Exploratory analyses testing whether less positive and more negative emotional states were experienced by anxious and avoidant individuals particularly when facing stress might offer some additional insight. While attachment orientations were not predictive of emotional experiences as associated with stress, both anxious and avoidant orientations predicted stronger negative emotional responses in times of stress, which could indicate that attachment effects manifest themselves specifically in stressful situations [78] and in the form of more negative emotional responses to stress [79–81]. Future studies may want to test and confirm this possibility.

The hypothesized associations between attachment orientations and emotional inertia did not receive support (H2a, H2b). The results for anxious individuals contrast with this prediction, suggesting that these individuals experienced less stable affect, as reflected by lower positive emotional inertia. Taken together, the data yield partial support for H1a and H1b, and no support for H2a and H2b.

The results for hypothesis H1a are largely consistent with the notion that anxiously attached individuals tend to perceive negative emotions as congruent with their attachment goals to get attention, and they may seek to sustain them [82], because signs of weakness and neediness can sometimes elicit attachment figures’ attention and care [31]. Hyperactivating strategies like rumination, used by anxiously attached individuals, may intensify negative emotions and overt displays of distress [83], hostility, and sadness [1]. Furthermore, a possible explanation for the experience of less positive and more negative affect might be a higher reactivity and less adequate recovery from negative affect [2]. The finding that the differences in affect reports associated with attachment anxiety were also enhanced in times of momentary stress, is consistent with such a possibility.

The hypothesis H1b that individuals with an avoidant orientation were expected to experience lower levels of positive and higher levels of negative affect was only partially supported. According to previous research, avoidant individuals adopt deactivating strategies that allow distancing from distress-eliciting events [1]. This may cause them to avoid noticing– or even reporting– their own negative emotional reactions. Avoidant individuals tend to divert attention from emotion related material and defend against the conscious experience of unpleasant emotions [11] by denying or suppressing them [84], which may explain why avoidant individuals in this study experienced more positive affect. Therefore, considering the possibility that avoidants suppress their negative emotions, it would seem possible that they would only report higher positive experiences but not negative ones. Moreover, prior studies have also shown that suppression resulted in more positive behaviors (e.g. smiling, laughing) [63], which is in line with the findings of the current study. Individual and situational differences may also help explain these results. Avoidant individuals experience activation of attachment-related worries only under conditions of high cognitive load [84]. Specifically, in severe and persistent stressful conditions imposing increasing demands on their cognitive system, avoidant individuals exhibit high levels of distress [85], which was confirmed by the current data showing that avoidant individuals experienced more negative affect at times when they reported stress. It could be that if individuals did not experience a prolonged stressful situation, the avoidant attachment system was not activated and therefore, attachment avoidance was not significantly associated with more negative emotions in general. Contextual factors such as current interactions with a partner, the attachment dynamics of the partner and a person’s current life situation (e.g. physical and psychological traumas) also modify the tendency that an avoidant individual has for deactivation of the attachment system and the experience of negative emotions [1]. Moreover, there is some evidence that, rejecting parents often discourage emotional display in their children and they do not teach them how to label and represent their emotions. As such, avoidant individuals are found to experience more negative emotions but fail to acknowledge them [86] and have greater difficulty in describing these emotions [87] relative to anxious individuals. Consequently, these conditions may lead to later difficulties in affect regulation [88].

In contrast to our hypothesis H2a, proposing that anxious individuals would show more inert positive and negative emotional states, we observed that anxious individuals showed less inert positive dynamics. In line with these findings, Koval & Kuppens (2012b) found that more vulnerable individuals had a greater drop in inertia levels when they anticipated a stressor suggesting that it is stress vulnerability underlying changes in emotional inertia. Considering that anxious individuals seek proximity to gain support and love [89] aiming at establishing intimacy and closeness [1] maybe this leads to more frequent emotional fluctuations (i.e., lower emotional inertia). Yet, another possibility is that in anticipation of, or during a stressor– like the transition to parenthood– individuals might change their daily activities, which might influence changes in moment-to-moment feelings [21]. Therefore, assuming that anxious individuals would exhibit higher inert emotional experiences might not be true in the first place because of their hyperactivating strategies during stress. It is important to emphasize, however, that although the interaction effects were highly significant, the simple slopes did not suggest significant autoregressive associations of positive affect for individuals with a comparatively anxious attachment style (relative to the sample mean in anxious attachment, which reflects intermediate levels of attachment anxiety). Individuals high in avoidance did not report more inert positive or negative emotions as was predicted by hypothesis 2b. Thus, they may report less negative emotion in daily life and show a faster recovery– lower levels of inertia– when they experience them, in an effort to emotionally distance themselves from distress-eliciting events, which could cause proximity-seeking and lead to their partner’s rejection [1]. These findings are in line with previous findings, which have shown that suppression– an emotion regulation strategy preferentially used by avoidant individuals [33, 78] is linked to restricted emotional flexibility [57, 63]. Suppressing feelings that imply vulnerability, results in ignoring important information about stressful situations and as such they did not show inert negative emotional experiences. In addition, another consideration is that the difficulty in regulating their emotions makes avoidant individuals to keep anger and resentment alive internally while attempting not to express them externally [78], which we suggest it needs further exploration. We also suggest that the fact that insecure individuals appear to respond stronger with negative emotions to stress might point to more instable affect [78–81]; maybe more negative affect inertia would emerge after major stress experiences, which should be tested in future studies.

One consideration is that priming thoughts of a supportive attachment figure and cognitive models of self and others lead people high on anxiety or avoidance to behave like more secure individuals [90]. Individuals with avoidant workings models when they are in stressful situations and their romantic partners support them with unsolicited support, are rated as more calmed [91]. This indicates that such persons can benefit from support, which can in turn lead them to experience less negative affect and less inert dynamics because their partner’s support buffered their attachment-related concerns. In our sample, couples were relatively happy expecting their first child, which means that they might have encountered more supportive and positive emotional experiences. In this line of reasoning, anxiously or avoidantly oriented individuals are less inclined to feel and behave according to their insecure working models when they are involved in more committed relationships [92] or are more dependent on their partner [93]. For instance, when they encounter stressful situations these individuals are less likely to react in insecure ways when their partner buffers their attachment related concerns, helping to actually experience less negative affect [94].

One question that deserves attention is how affect reactivity and inertia go together and whether they can co-exist, especially when people experience and report stress. Our results show that stress predicts less positive and more negative affect generally. However, in both anxious and avoidant individuals reported feeling stressed predicts more negative but not less positive affect, raising the question of whether some people are more stress-reactive and what mechanisms are activated after a stressful experience that they might have failed to cope and regulate their emotions.

## Limitations

The results reported in this study should be appreciated with caution due to several limitations. First, due the to the correlational nature of the analyses, causal inferences among attachment insecurity and emotion or emotional inertia cannot be drawn with certainty. Second, participants were healthy and involved in stable, relatively happy romantic relationships, expecting their first child. It is therefore unclear whether these results can be generalized to other relationships in the broader population. Third, the current study focused on attachment insecurity and its effect on the experience of affect and its over time dynamics. Several other factors that potentially influence affect experience and dynamics have not been considered in this study (e.g. felt intimacy, current partner interactions). Fourth, we assessed momentary affect four times a day and reported analyses using three time points (we did not include overnight estimates), and this might be too coarse a sampling grid to capture everyday emotional fluctuations. Considering this, it is reasonable to anticipate relatively modest cross-level interaction effects. Therefore, a more fine-grained assessment of momentary affect fluctuations may be necessary to capture fluctuations in affect inertia [57] or momentary emotional experiences.

## Conclusion and future directions

Despite the limitations, our study advances the current understanding of how emotions and emotional inertia are associated with attachment anxiety and avoidance, especially when individuals feel stressed. Furthermore, studying momentary fluctuating affect offers complementary insight into the emotional functioning of individuals with different attachment orientations. Further research is needed to better understand the role of attachment in the experience of emotions, their sustainability through time and their impact on subsequent functioning and social interactions. It still needs to be clarified why affective experiences are more or less inert in some people than in other and how attachment orientation and felt stress potentially affect these experiences. The current study examined only the subjective feeling component of emotions. However, emotions are considered to also involve changes in behavior, and physiology [95]. Therefore, future studies should focus on temporal dynamics of emotions as the outcome of multiple factors and processes. Additional investigation on the role of gender and attachment in relation to emotions is warranted. The study of individual differences in attachment orientations, the experience of emotion and emotion dynamics, is likely to contribute to our understanding of why close relationships vary in both their quality and their interpersonal nature.

## Data Availability

A final dataset used for the analyses of this manuscript can be found here: 10.17605/OSF.IO/Y3BX8.

## Abbreviations

a: Cronbach’s coefficient of internal consistency
b: Beta coefficient
*p*: Probability value
r: Pearson correlation coefficient
CI: Confidence interval
ECR: Experiences in close relationships
ICC: Intraclass correlation coefficient
LA: Lagged affect
MLM: Multilevel modeling
NA: Negative affect
PA: Positive affect
SD: Standard deviation
