# Correction: Attachment insecurities, emotion dynamics and stress in intimate relationships during the transition to parenthood

**DOI:** 10.1186/s40359-024-01775-w

**Published:** 2024-05-16

**Authors:** Georgia Kouri, Nathalie Meuwly, Marianne Richter, Dominik Schoebi

**Affiliations:** Department of Psychology, University of Fribourg, Rue de Faucigny 2, Fribourg, 1700 Switzerland

**BMC Psychology (2024) 12:200**

10.1186/s40359-024-01686-w

Following publication of the original article, it came to the author’s attention that the following source of funding had erroneously not been acknowledged in the declarations of the article: “This research was funded by the Swiss National Science Foundation grant number 100014_175620.” The article has since been updated with this acknowledgement. The authors thank you for reading and apologize for any inconvenience caused.

